# Ultrasound-Guided Regional Anesthesia Using a Mixture of Dexamethasone, Dexmedetomidine, and 0.2% Levobupivacaine for Bilateral Breast Cancer Surgery Under a Spontaneous Breathing Opioid-Free Anesthesia: A Case Report

**DOI:** 10.7759/cureus.58394

**Published:** 2024-04-16

**Authors:** Fabrizio Falso, Roberto Giurazza, Clotilde Crovella, Rosanna Carmela De Rosa, Antonio Corcione

**Affiliations:** 1 Department of Critical Care, AORN Ospedali dei Colli, Naples, ITA; 2 Department of Woman, Child, General and Specialized Surgery, Università degli Studi della Campania "Luigi Vanvitelli", Naples, ITA; 3 Department of General and Specialized Surgery, AORN Ospedali dei Colli, Naples, ITA

**Keywords:** awake breast surgery, parasternal block, serratus anterior plane (sap) block, erector spinae plane (esp) block, spontaneous breathing sedation, opioid-free analgesia, dexmedetomidine, dexamethasone, ultrasound-guided regional anesthesia, synchronous bilateral breast cancer

## Abstract

Breast cancer is unfortunately the most common cancer in women, although survival rates have greatly increased in recent years. Breast surgery can be very aggressive and therefore highly painful, leading to high rates of acute postsurgical pain and chronic pain. In addition to general anesthesia (GA), ultrasound-guided regional anesthesia (RA) is sometimes performed to help reduce acute postoperative pain and consumption of opioids. Although effective, the main limitation of fascial plane blocks is that they require high volumes of local anesthetics, carrying the risk of local anesthetic systemic toxicity. In this article, we present the case of a 41-year-old woman, who refused GA and was successfully operated on for bilateral breast cancer, under a spontaneous breathing opioid-free sedation and ultrasound-guided RA, based on only 0.2% levobupivacaine with the addition of dexamethasone and dexmedetomidine as adjuvants. Despite this, postoperative analgesia lasted for more than 48 hours, and the patient did not require additional analgesia or opioids.

## Introduction

According to the American Cancer Association and Cancer Research United Kingdom, after skin cancer, breast cancer is the most common malignancy in women, accounting for almost one-third of all female cases, with a trend toward an increasing annual incidence [1]. Life expectancy and survival rates in women diagnosed with breast cancer have dramatically improved over the last 20 years, due to screening campaigns, better diagnosis, more conservative surgical techniques, and personalized targeted medical treatments [1]. Moreover, breast reconstruction - with the implantation of a tissue expander or a definitive breast prosthesis - can be performed immediately after breast demolitive surgery, reducing the impact on patients’ psychology, and improving their quality of life.

Breast surgery - especially total and radical mastectomy with axillary lymph node dissection (ALND) - can be associated with moderate to severe acute postsurgical pain, requiring high doses of painkillers, including opioids with their associated detrimental side effects. It can also negatively affect postoperative outcomes, with higher rates of complications, higher sympathetic tone, more patient distress, and longer hospital stays, with total higher costs [2]. Acute postoperative pain has been identified as one of the main causes of chronic postoperative pain after breast surgery and should be adequately addressed to avoid such a debilitating consequence.

Worldwide, in most cases, oncological breast surgery is performed under general anesthesia (GA), with postoperative continuous intravenous analgesia [3]. In order to facilitate optimal pain control, with early mobilization and fast discharge of the patient, the Enhanced Recovery After Surgery (ERAS) and PROcedure SPECific postoperative pain managemenT (PROSPECT) guidelines on breast surgery highly recommend the use of multimodal analgesia, supporting the use of local anesthetic (LA) infiltration or regional anesthesia (RA) techniques, with the adoption of opioid-sparing and opioid-free regimens. Importantly, opioids should be used only as a rescue medication, if non-opioid analgesics and regional analgesic techniques do not provide sufficient pain control [3,4]. Opioid-free anesthesia (OFA) has been proven to be feasible, effective, and advantageous in different surgeries, including breast surgery [5]. 

After the first description in 2011 by Blanco et al. [6] of the pectoral nerve blocks (PECS 1 and PECS 2), a new era of RA was started even for breast surgery, with the description of different other techniques, including serratus anterior plane block (SAPB), erector spinae plane block (ESPB), paravertebral block (PVB) and parasternal block (PSB). Being fascial plane blocks, these RA techniques are performed using ultrasound guidance and require the injection of high volumes of LA (e.g., 20-30 mL of 0.5% ropivacaine or 0.375% levobupivacaine), allowing the unzippering of the fasciae and the spread of LA over different nerves, obtaining the coverage of a widespread area of the thoracic wall.

Since these are high-volume fascial plane blocks, the anesthesiologist must always be aware of the maximum dose of LA that can be administered to that patient so, as not to incur local anesthetic systemic toxicity (LAST). This is especially true in case a surgical procedure requires multiple different fascial plane blocks in the same patient, such as in the case of bilateral breast surgery. In such cases, the anesthesiologist must wisely adapt the LA concentration, with the use of adjuvant drugs (dexamethasone, clonidine, dexmedetomidine, etc.), without sacrificing the volume of the injectate and without excessively lowering the LA concentration under the minimum local anesthetic concentration (MLAC), i.e., under the minimum effective anesthetic/analgesic concentration. There is convincing evidence that the combination of perineural dexamethasone and dexmedetomidine can increase the potency and duration of the LA administered, as demonstrated by Pullano et al. and other authors [7].

## Case presentation

A 41-year-old woman came to our attention with a diagnosis of bilateral breast cancer and was scheduled to receive a left total mastectomy with prosthesis implantation and left axillary sentinel lymph node biopsy (SLNB), as well as a right upper external quadrantectomy with right axillary SLNB. An intraoperative extemporaneous histopathological examination was planned for all surgical specimens.

The patient's past medical history was irrelevant, except for essential arterial hypertension (treated with felodipine 5 mg daily), grade 1 obesity, and a reported allergy to valsartan. Her height was 160 cm and her weight was 81 kg (BMI was 31.6 kg/m^2^). The patient denied smoking cigarettes or using recreational drugs or alcohol. She had already undergone previous surgeries under LA and GA, without reporting any adverse events. She was attributed a class two risk, according to the American Society of Anesthesiologists (ASA) classification. When signing informed consent for anesthesia, the patient refused GA with mechanical ventilation, but she gave consent for RA and sedation.

On the morning of surgery, in the pre-anesthesia room, the patient received the following premedication intravenous (IV) drugs: midazolam 3 mg, pantoprazole 40 mg, dexamethasone 8 mg, ondansetron 8 mg and magnesium sulfate 40 mg/kg. She also received preoperative antibiotic prophylaxis with 2 g of cefazolin IV within 30 minutes of skin incision and preemptive analgesia with 1 g of acetaminophen and 30 mg of ketorolac IV.

Due to the patient’s refusal of GA, despite the complexity of the surgical case which would have involved both breasts and both axillae, we performed RA on multiple sites and both sides of the chest wall, in order to provide complete intraoperative analgesia, as well as long-lasting postoperative pain control.

All RA procedures were carried out with a complete aseptic technique, after skin disinfection with 2% chlorhexidine, and under ultrasound guidance (BK medical™, BK5000™) with a high-frequency linear probe. A Pajunk™ UniPlex™ 22G 100 mm single-shot needle was used. Patient vital signs were continuously measured. We prepared a local anesthetic mixture of 0.2% levobupivacaine + dexmedetomidine 0.6 μg/mL + dexamethasone 0.06 mg/mL, which was used for all the injections.

**Figure 1:**
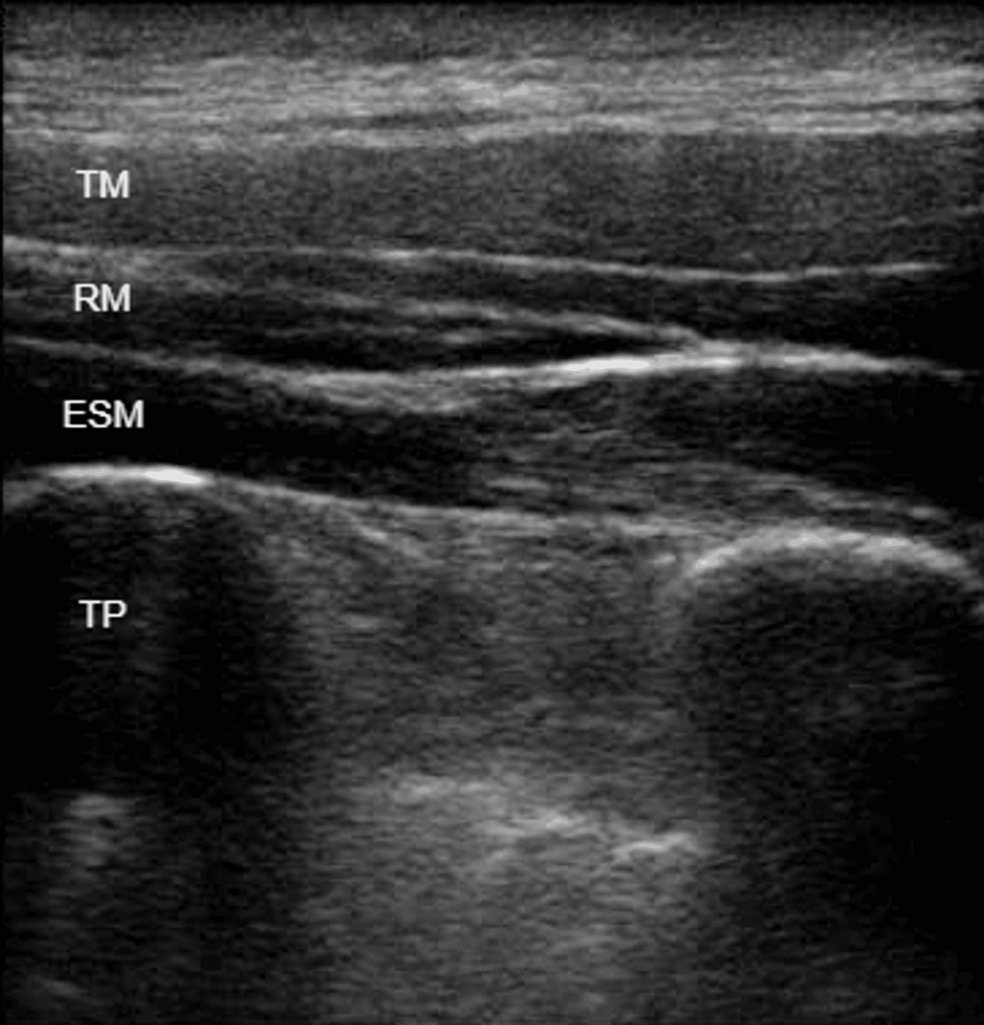
Erector spinae plane block (ESPB). TM: trapezius muscle. RM: rhomboid muscle. ESM: erector spinae muscle. TP: transverse process.

**Figure 2:**
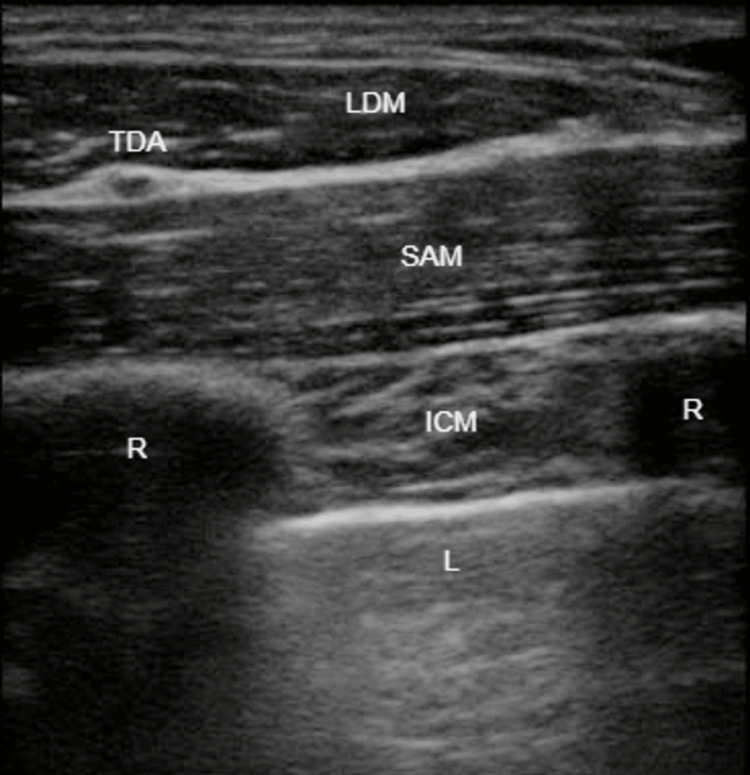
Serratus anterior plane block (SAPB). LDM: latissimus dorsi muscle. SAM: serratus anterior muscle. TDA: thoracodorsal artery. ICM: intercostal muscles. R: rib. L: lung.

**Figure 3:**
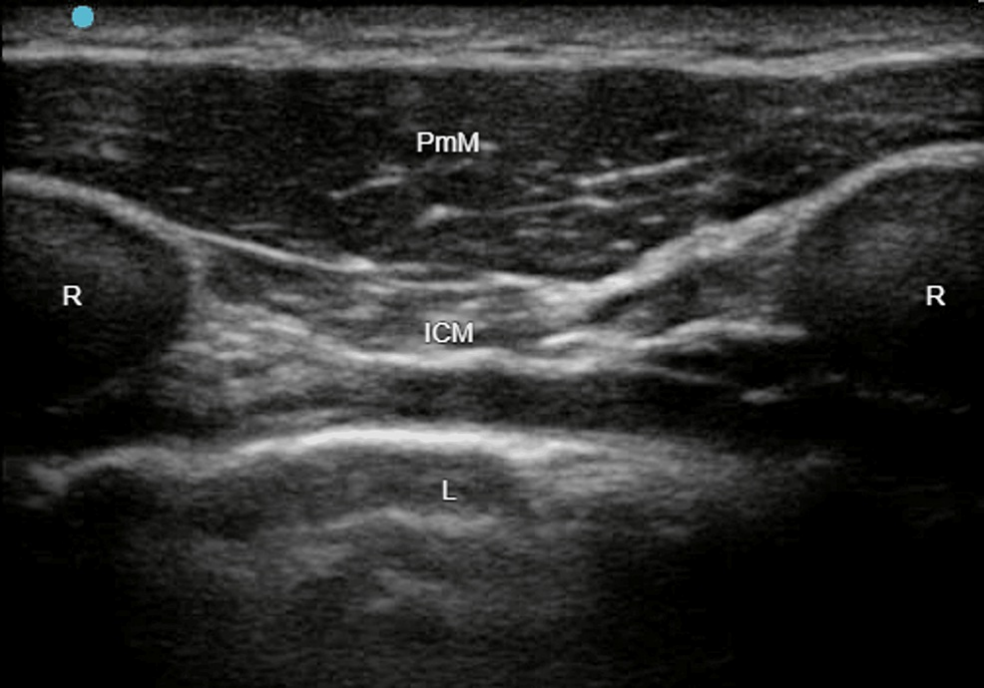
Parasternal block (PSB). PmM: pectoralis major muscle. ICM: intercostal muscles. R: rib. L: lung.

On the left side, we performed an ESPB at the level of the fourth thoracic transverse process (T4), as well as an SAPB and a PSB, with the injection of 20 mL, 20 mL, and 10 mL of LA mixture, respectively. On the right side, we performed a SAPB with the injection of 20 mL of LA mixture (Figures 1-3).

All the RA procedures were uneventful. The total volume of the LA mixture administered was 70 mL; therefore, the total doses of drugs administered were 140 mg, 42 μg, and 4.2 mg for levobupivacaine, dexmedetomidine, and dexamethasone, respectively. Being levobupivacaine's maximum dose of 2.5 mg/kg, we stayed under the toxic dose threshold.

Thirty minutes after the injections of RA, the sensory block was tested using a skin pinprick test, which confirmed successful anesthesia from the first to the seventh thoracic (T1-T7) metamer. Then, the patient was moved to the operating theater.

Since the surgeons estimated the surgery to last at least four hours, the patient could have experienced significant positional discomfort during the operation. Therefore, an IV propofol target-controlled infusion (TCI) was started with an effect-site concentration of 0.8-1.2 μg/mL and a Venturi mask was applied with a FiO_2_ of 40%.

Electrocardiogram (ECG), heart rate, noninvasive blood pressure (NIBP), respiratory rate, peripheral oxygen saturation (SpO_2_), and end-tidal carbon dioxide (EtCO_2_) were continuously monitored during the operation. A urinary Foley catheter was temporarily placed to drain urine and monitor its output, with prompt removal immediately after the end of surgery. The depth of anesthesia/sedation was continuously monitored with the analysis of Bispectral Index™ (BIS™) Monitoring System (Medtronic™).

The intraoperative extemporaneous histopathological examination of the left SLNB was negative. Unfortunately, it was positive for the right SLNB, so our surgeons had to proceed to the right ALND.

The complex surgical operation was uneventfully completed in five hours and did not lead to significant blood loss. We report one episode of sinus bradycardia (HR <45 bpm), that was promptly and effectively treated with atropine 0.01 mg/kg IV. Besides this, vital signs were always stable and BIS values were constantly between 50 and 70 throughout all the duration of surgery. The patient did not experience any discomfort or pain during the operation, and she did not receive any additional drugs, neither opioids nor additional rescue analgesia. A total of 1,500 mL of balanced crystalloids (1,000 mL of Lactated Ringer’s and 500 mL of electrolyte solution) were administered, with a total urine output of 350 mL.

At the end of the surgery, after awakening, the patient was calm and did not complain any pain at rest and during active motion of both upper arms (numerical rating scale, NRS, = 1/10), except during the positioning of the compressive breast binder (NRS = 3/10).

Postoperative analgesia prescription included IV acetaminophen 1 g every eight hours, with IV ketorolac 30 mg as a rescue analgesic (maximum 90 mg daily), in case NRS was >3. The patient was monitored in the recovery room for 30 minutes, during which she didn’t present any adverse events (nausea/vomiting, shivering, pain, etc.) and then was escorted to her hospital room.

Two hours after the end of surgery, she started drinking clear fluids and two hours later she ate a light dinner, without complaints of nausea or vomiting, and started walking in the ward. Pain NRS scores were monitored at 6, 12, 24 and 48 hours after surgery and were always ≤2/10. In the days following surgery, the patient did not experience any adverse events or complications, and she was discharged home on the fourth postoperative day in good general condition and pain free. Our patient gave written informed consent for the publication of this case report.

## Discussion

In this case report, we describe the feasibility of even complex bilateral breast surgery with different combinations of RA techniques with dexmedetomidine, dexamethasone, and low LA. Despite being the most common type of anesthesia used worldwide for breast surgery, GA does not come without risks, including respiratory, cardiovascular, neurological, infectious, and metabolic complications. These can significantly increase the length of hospital stay and may require additional tests, consultations, and drugs. Therefore, they can skyrocket overall costs and increase patients' morbidity [8].

In most cases, RA for breast surgery is not used as the primary anesthesia technique, but in combination with GA to ensure better postoperative pain control. The rates of cardiovascular and pulmonary complications associated with RA are way lower than those associated with GA [9] and the risk of LAST can be minimized by ultrasound guidance, continuous aspiration-injection technique, and the accurate prescription of LA dose and concentration.

From a perspective view, the advantages and implications of RA for breast surgery are even bigger. Indeed, there is emerging and convincing evidence that the avoidance of GA and opioids could influence oncological outcomes [10]. Some studies comparing RA and GA have shown a lower recurrence risk of breast cancer in patients treated with RA, probably through natural killer cells and T-lymphocytes. Studies have shown that RA may offer some benefits in terms of cancer recurrence, namely a direct antitumoral effect of amide LA, reduced opioid use with their adverse effects on cancer recurrence, and a reduction of the stress response and its associated immunosuppression [10].

Compared to GA, spontaneous breathing sedation has a lot of advantages, including the absence of neuromuscular blocking agents (NMBA), endotracheal intubation, and mechanical ventilation, with obvious favorable consequences on postoperative outcomes, primarily very low or minimal impact on pulmonary function and fairly lower risk of postoperative pulmonary complications (PPC), including multidrug-resistant ventilator-associated pneumonia [11].

Thanks to its optimal intraoperative and postoperative analgesia, RA can be successfully used for breast surgery, in combination with sedation, without the need for GA. This allows us to avoid cardiopulmonary complications associated with GA, while at the same time reducing postoperative pain scores and opioid use with overall beneficial effects [9,12].

Currently, the PROSPECT guidelines recommend preoperative thoracic paravertebral block (TPVB) as the first-choice analgesic technique in breast surgery [4], since it has been shown to be associated with lower postoperative pain scores, lower opioid consumption, lower postoperative nausea and vomiting (PONV) and a shorter hospital stay, compared with GA with systemic opioid-based analgesia alone. However, PVB is an invasive procedure, with a risk of significant complications, such as pneumothorax, pleural puncture, epidural spread, hypotension, and accidental intravascular injection, as well as the risk of bleeding in patients using antithrombotic medications [4].

Currently, besides PVB there are other RA techniques available for analgesia and anesthesia of the thoracic wall, therefore suitable and appropriate for breast surgery, including PECS blocks, SAPB, ESPB, and PSB [13]. Under ultrasound guidance, these procedures are safe and effective and are increasingly preferred by anesthesiologists to TPVB, since they do not carry the notable risk of pneumothorax [6]. Their application in breast surgery reduces acute pain scores, the use of opioids, and the incidence of chronic postsurgical pain [14].

Being fascial plane blocks, they require an injection of a considerable volume of long-acting LA, e.g., approximately 20-30 mL of 0.375% levobupivacaine for ESPB and SAPB. Hence, the anesthesiologist who performs these blocks must always be aware of the risk of LAST and wisely adjust LA concentrations, sometimes with the use of adjuvants (i.e., clonidine, dexmedetomidine, and dexamethasone). Besides dexamethasone, which has a well-established role as an adjuvant, dexmedetomidine has recently shown interesting properties, especially in combination with intrafascial or IV dexamethasone, increasing the potency of the mixtures, as well as the duration of sensory block [7,15]. Anyways, the volume of the injectate should not be reduced and the LA concentration should not be lowered under the minimum local anesthetic concentration (MLAC) for efficient analgesia.

In the case we presented, the combination of ESPB, SAPB, and PSB on the left side could seem redundant, since SAPB and PSB theoretically could have been sufficient to anesthetize the area of left total mastectomy. The association of ESPB was implemented to enhance the potency and coverage of SAPB and PSB, as other authors have already pointed out. Indeed, the association of fascial plane blocks proximal to nerve origin, such as ESPB and PVB, with blocks more distal from nerve origin, such as PECS, SAPB, and PSB, has been shown to be effective and feasible in awake breast surgery with reconstruction by several studies, including case reports, case series and randomized controlled trials [12,16-19]. Such a combination of nerve blocks might be beneficial for two main reasons: (1) there is a spread of LA from the erector spinae plane and from the paravertebral space to the epidural space, thus on ventral and dorsal nerve roots; (2) there is a block of small fibers (A-delta and C) at two different levels, proximal and distal, resulting in a complete block of nerve conduction and allowing lower concentrations of LA for complete nerve block.

Based on all that has been exposed thus far, our case report should be considered innovative for different reasons. First, for such a complex case of breast cancer surgery, respecting our patient’s will, we did not opt for conventional opioid-based GA, but we offered our patient ultrasound-guided RA with opioid-free spontaneous breathing sedation, without the need for curarization and mechanical ventilation and with an extremely fast recovery. Second, we performed multiple fascial plane blocks, with a significantly high total volume (70 mL), using a very low concentration of levobupivacaine (0.2%), without incurring in LAST. Despite this, our blocks were not only analgesic but also anesthetic since the whole surgery was conducted without the need for rescue analgesia. Third, due to the addition of adjuvants (dexmedetomidine and dexamethasone in the LA mixture), our patient experienced extraordinarily long analgesia, which lasted for more than 48 hours, despite the low concentration of the LA mixture, without the need for rescue analgesics (including opioids) and any significant side effects.

Other authors have pointed out how the combination of such adjuvants (dexmedetomidine and dexamethasone) in the LA mixture can prolong the potency and duration of analgesia, although not in breast surgery [20]. We hope that, in the future, further studies will corroborate our findings and international guidelines will endorse even more RA in lieu of GA for breast surgery, for the sake of patient wellness, less postoperative pain, less associated cardiopulmonary complications, enhanced recovery, and better cancer outcomes.

## Conclusions

Our study, although only a case report, shows how even complex reconstructive breast surgery, such as bilateral breast cancer, can be successfully accomplished using well-studied and appropriate RA techniques, without the need for GA and opioids. The addition of adjuvant drugs, such as dexmedetomidine and dexamethasone, allows the reduction of LA concentration under limits otherwise unimaginable, as well as long-lasting postoperative analgesia, without side effects and with fast recovery and discharge home.
